# Community perceptions of the implementation and impact of an intervention to improve the neighbourhood physical environment to promote walking for transport: a qualitative study

**DOI:** 10.1186/s12889-018-5619-y

**Published:** 2018-06-08

**Authors:** Emma J. Adams, Lauren B. Sherar

**Affiliations:** National Centre for Sport and Exercise Medicine, School of Sport, Exercise and Health Sciences, Loughborough University, Loughborough, LE11 3TU UK

**Keywords:** Walking, Transport, Pedestrian, Built environment, Physical activity, Implementation

## Abstract

**Background:**

Using community engagement approaches to develop and deliver interventions targeting small-scale physical environmental improvements in neighbourhoods is a potential strategy for increasing walking for transport. This study aimed to qualitatively assess community perceptions of the implementation and impact of the Fitter for Walking (FFW) intervention, which encouraged communities to work together to improve the street environment on local routes and promote walking for transport.

**Methods:**

From 155 FFW community projects, nineteen were selected to take part in a focus group/interview using specified criteria: geographical area; level of community involvement; intervention activities; and project progress. Participants were invited to take part via the project coordinator or lead member of the community group. A written guide was used to initiate and direct discussions through key topics. Deductive and inductive approaches were used to analyse the data and identify key themes relating to the barriers and facilitators for implementation and the perceived impact of the intervention.

**Results:**

Fourteen focus groups and five interviews were conducted with 86 community members. Themes were identified in relation to barriers (poor area reputation and regeneration areas; engaging the local community; and working with local authorities) and facilitators (provision of a coordinator/facilitator; strong local partnerships; and using a range of communication and engagement activities) for programme implementation. Participants perceived the main impacts to be improved physical and social environments. Increases in walking for transport were rarely specifically commented on, but participants did report increased street use.

**Conclusions:**

Community perspectives provided important insights into the barriers and facilitators for the implementation of the FFW intervention and its’ potential impacts. Using community engagement approaches can lead to perceived improvements in the physical and social environment resulting in increased street use, which may lead to increases in walking for transport in the longer-term. Recommendations are provided for researchers, practitioners and policy makers in planning and delivering future interventions. Future research should determine optimal implementation strategies, investigate the relative importance of improving physical environments, social environments and using individual behaviour change strategies, and determine how physical and social environments interact to maximise intervention impact on walking for transport.

**Electronic supplementary material:**

The online version of this article (10.1186/s12889-018-5619-y) contains supplementary material, which is available to authorized users.

## Background

Despite the well-known benefits of physical activity for health and well-being, levels of participation in England remain low [1]. High levels of physical inactivity and resulting non-communicable disease place a significant burden on society through costs to the economy and healthcare [2]. Strategies which effectively increase physical activity are urgently needed to improve health and reduce this burden.

Walking can benefit health [3–5], is convenient, does not require special skills or equipment and can be easily incorporated into daily routine [6]. It can be undertaken for recreational or transport purposes. Whilst recreational walking remains one of the most popular activities [7], levels of transport-related walking are declining. In 1995/1997, 27% of short journeys were undertaken on foot compared to 22% in 2015. The average distance walked (miles per person per year) also decreased, by 15 miles, in the same period [8]. Promoting walking for transport is therefore a potential target for strategies aiming to increase physical activity [9]. Replacing passive modes of transport with active modes, such as walking, also has the possibility to address other goals, such as reducing traffic congestion and greenhouse gas emissions, and improving air quality [10].

The physical environment has been recognised as an important factor influencing physical activity (including walking) in ecological models [11]. Attributes of the physical environment are known to be associated with walking for transport [12–16]. For example, in a recent study in England, walking for transport was positively associated with supportive infrastructure, availability of local amenities and general environment quality [12]. In a pooled analysis of data from the USA, Australia and Belgium, a walkability index, including residential density, accessibility, proximity to destinations and aesthetics, showed positive associations with walking for transport [15]. Whilst there has been some variation in the environmental attributes assessed in different studies, it has been hypothesised that interventions which improve physical environment attributes in neighbourhoods may increase walking for transport and overall levels of physical activity. In addition, targeting the neighbourhood physical environment for improvements has the potential to increase the reach of interventions compared to individually focussed interventions, due to the numbers of individuals exposed to the environment [17].

To date, many published environmental interventions have focused on large scale infrastructural changes such as new roads, new or improved public transport systems, or substantive changes to walking and cycling infrastructure [18–23]. Only a few interventions have been reported which have implemented smaller-scale improvements to the physical environment (such as improved lighting, improvements to road crossings, improved continuity of footpaths, traffic calming measures e.g. speed bumps and improved aesthetics of the route e.g. landscaping) in neighbourhoods [17, 24, 25]. Installing or improving lighting, redesigning streets and making improvements to street aesthetics have been identified as effective intervention strategies in small geographic areas [17]. These types of interventions may be of lower cost and more rapidly implemented, thus they may offer an important strategy for improving the neighbourhood environment to promote walking for transport.

Community engagement can be defined as the “direct or indirect process of involving communities in decision making and/or in the planning, design, governance, and delivery of services using methods of consultation, collaboration, and/or community control” [26]. Using community engagement approaches to design and deliver interventions can help improve health and well-being and reduce health inequalities [27, 28]. They can also lead to increases in social capital, community capacity building and empowerment of community members [28, 29]. There is a paucity of published interventions that have used a community engagement approach to initiate and implement small-scale environmental improvements, with the aim of improving the local neighbourhood walking environment and increasing walking for transport. The authors are aware of only one study, conducted in the US, which has reported this approach [30]. In this study, residents from a local community were recruited to work in partnership with community-based organisations, public health practitioners and researchers. Barriers and facilitators for walking were identified and informed the development and delivery of intervention activities. These included: improvements to a staircase on a walking route; campaigns to restrict on-street parking, improve traffic signals, introduce speed monitors on busy streets and enhance safety at busy road junctions; the formation of walking groups; and promotion of walking opportunities in the local area. Significant increases in mean time spent walking were observed (from 65 to 109 min per day) as well as significant increases in the proportion of participants undertaking 150 min moderate-intensity physical activity per week (from 62 to 81%). However, this was only measured in walking group participants rather than at the community level. In addition, whilst previous studies have considered the barriers and facilitators to taking part in community-based walking interventions as a participant [10], none have explored the barriers and facilitators from a community perspective when they are personally involved in designing and/or delivering intervention activities. Furthermore, community perceptions of the impact of such an intervention have not been explored. Understanding the perspectives of the communities involved in intervention implementation and their perceptions of intervention impact is valuable for those developing and designing future interventions which aim to improve the local environment to promote walking for transport. It also aids understanding of the role and impact of community engagement approaches in this type of intervention.

The Fitter for Walking (FFW) intervention was delivered in deprived neighbourhoods across England where physical activity levels were reported to be low. Community groups and local residents were consulted and recruited to support the development and delivery of the intervention which aimed to improve the street environment on local routes to key destinations, such as shops, public transport, workplaces, schools, leisure facilities and entertainment venues, to promote walking for transport. The aim of this paper was to qualitatively evaluate community perceptions of the barriers and facilitators for intervention implementation and the perceived impact of the FFW intervention.

## Methods

### ‘Fitter for Walking’ intervention

FFW was delivered between August 2008 and March 2012 and was managed by a third sector organisation (referred to as the lead organisation). The intervention aimed to: improve the local neighbourhood walking environment; encourage communities and local residents to work together to promote walking; and increase the number of people walking on specific local routes to key destinations targeted for environmental improvements. Twelve local authorities (LA) from five regions of England were recruited as partners in the project (Table 1) and a full-time coordinator was employed in each region by the lead organisation to work with communities and coordinate intervention activities. Community groups were recruited using reactive approaches (community groups or local organisations contacted the coordinator) and proactive approaches (coordinators contacted community groups). The coordinators worked with the groups to identify a local route or area on which to focus the project. Members of the group and residents from the wider community were consulted to identify barriers to walking on the specified route using a Community Street Audit [31] or at local group meetings. Following the consultation, coordinators made recommendations to communities, LAs and partners regarding how barriers to walking might be overcome which informed the activities that were undertaken during the intervention. Overall, 155 community projects took place during the intervention period with one community group registered with FFW per project. Registered community groups included: tenants’ and residents’ associations, churches, local interest groups (e.g. allotment associations, wheelchair users’ groups or ‘Friends of’ groups), specific ethnic groups, community centres and schools. Members of the community groups and local residents were encouraged to get involved in planning and delivering intervention activities. Three types of activities were implemented: (1) local authority-led infrastructural changes (e.g. new street lighting, dropped curbs, removal of street furniture such as bollards or railings); (2) community-led environmental changes (e.g. bulb planting, street cleaning); and (3) coordinator- or community-led promotional and awareness raising activities (e.g. led walks to increase awareness of a newly improved route, or street parties). The types of activities implemented in each project varied and not all types of activity were implemented in all projects. An evaluation of the implementation of the intervention from the coordinators’ perspectives, and the impact of the intervention on walking levels (route use), have been reported previously [32, 33].

**Table 1:** Fitter for Walking communities where focus groups and interviews were conducted

Region	Local Authority (LA)	Community Project	Focus Group/ Key Informant Interview (date)	Number of participants	Males n	Females n
London	London Borough of Barking & Dagenham	Marks Gate Older People’s network	Focus Group (Oct 10)	7	4	3
	Redbridge Borough Council	Seven Kings & Newbury Park Residents’ association	Interview (Oct 10)	1	0	1
North East	Gateshead Council	Gateshead Jewish Nursery (Bensham)	Interview (June 10)	1	1	0
		Local Felling Residents	Focus Group (Mar 11)	4	2	2
	Newcastle City Council	Friends of St Lawrence Park (Byker Link)	Focus Group (June 10)	5	2	3
		Trinity Gosforth	Focus Group (Mar 11)	3	0	3
	Sunderland Council	Plains Farm and Humbledon Residents’ Association	Interview (Mar 11)	1	1	0
North West	Blackburn with Darwen Council	Taylor Street	Focus Group (Nov 10)	6	1	5
		Empire Theatre	Interview (Nov 10)	2	0	2
	Bolton Council	Hallith Wood / Pixmore Paths	Focus Group (June 11)	7	2	5
		Our back field (Larkfield Grove)	Focus Group (June 11)	3	2	1
West Midlands	Dudley Metropolitan Borough Council	–	–	–	–	–
	Sandwell Metropolitan Borough Council	Friends of Thimblebrook Mill	Focus Group (June 11)	5	1	4
	Wolverhampton City Council	Lanesfield Tenants & Residents Association/Hilton Hall Management Association	Focus Group (Oct 10)	14	6	8
		Weddell Wynd Residents	Focus Group (Oct 10)	4	2	2
Yorkshire	Doncaster Metropolitan Borough Council	Friends of Hexthorpe Flatts Park	Focus Group (Apr 11)	5	2	3
		Latin Gardens/Emley Drive Area Tenants & Residents Association	Focus Group (Nov 10)	5	1	4
		Friends of Martinwells Lake/Edlington Royal Tenants & Residents Association	Focus Group (Nov 10)	6	3	3
	Rotherham Metropolitan Borough Council	Cliff Hills Community Action Group	Interview (Aug 11)	1	1	0
		Chinatown Tenants & Residents Association	Focus Group (Apr 11)	6	2	4
Total				86	33	53

### Data collection

Each coordinator kept an implementation log for their areas on a bespoke Microsoft Excel spreadsheet listing each of the community groups and recording details and progress for each project. In brief, this included items such as name of registered group, how the group was recruited, details of the target community (estimated size of population), main route/area targeted for intervention activities and intervention activities undertaken. These logs were updated and sent to the research team by the coordinators monthly. The research team reviewed the implementation logs at two different time points during the intervention (August 2010 and February 2011) with the specific aim to identify community projects which would be suitable for focus groups/interviews. The criteria for selection included: area (projects from different geographical areas were sought to represent a variety of different contexts across the five areas of England involved in the project); level of community involvement (projects were sought which had a range of local project partners and good engagement with the local community/residents); and, the types of intervention activities undertaken and progress made (projects had been involved with FFW for at least 12 months and had implemented a variety of environmental changes and/or promotional and awareness-raising activities). Based on these criteria, nineteen community projects were identified and approached to take part in the focus groups/interviews. All selected projects agreed to participate. Participants were invited to take part in the focus group discussion or interview by their coordinator and/or the lead member of the community group. Focus groups and interviews were arranged at a time to suit the participant(s) at a venue in the local community, for example the local community centre or library.

Focus groups and interviews were conducted by a female researcher (EJA). The purpose of the research was explained to participants and all participants gave written informed consent to take part before discussions commenced. An interview or focus group guide [see Additional file 1] was used to initiate and direct the discussions through key topics which included: project purpose and goals, roles and responsibilities of different stakeholders in the intervention, implementation of intervention activities, communication about activities, perceived impact of the project (including positive and negative (expected and unexpected) effects). The discussions lasted 45–60 min and were recorded with the participants’ agreement.

### Data analyses

All interviews and focus groups were transcribed verbatim by an independent administrator. Transcripts were read thoroughly to fully understand participants’ perspectives. Transcripts were then re-read and were initially coded in NVIVO Version 10 using a deductive process to collate findings from each of the focus groups/interviews into themes related to topics in the focus group/interview guide. An inductive approach was used to examine the coded data in more detail and to identify and organise themes relating to the barriers and facilitators for intervention implementation and perceived impact. Emergent themes related to the perceived impact of the intervention were also compared against the intervention aims, and unexpected impacts were identified. Data analyses were undertaken by EJA. Findings are supported with illustrative quotes, the source of which is identified using “FG” (focus group) or “Interview” and the year in which the FG or interview was conducted (e.g. FG, 2009).

Four criteria were used to ensure trustworthiness of the data: credibility; dependability; transferability; and confirmability [34, 35]. Credibility was established by: using recognised research methods; developing familiarity with the project context prior to conducting the focus groups/interviews through discussions with the local coordinator, using the implementation log, visiting the local area where environmental improvements had been undertaken or obtaining photographs of the improvements undertaken; the use of strategies to encourage participants to answer questions honestly, such as confirming there are no right or wrong answers, highlighting the independent nature of the researcher from the project, and ensuring the coordinator was not present during the focus groups/interviews; and using iterative questioning and probes to prompt more detailed and confirmatory information. Dependability was addressed by providing a full description of the study including intervention delivery and data collection methods (processes, participants and timelines for data collection). Transferability was addressed by providing details about the location and context of each project (Additional file 2). Finally, to minimise bias in the researcher’s interpretation of the data, confirmability was addressed through discussing emergent themes with another academic with expertise in undertaking qualitative research in the field of physical activity and public health.

## Results

Discussions were held with 86 participants (33 males and 52 females) from 19 community projects (14 focus groups and 5 interviews) (Table 1). Further details about each of the community projects including the registered group, start and end date of involvement in FFW, the project area or route, date of the street audit and intervention activities are provided in Additional file 2.

### Barriers and facilitators for intervention implementation

Participants in focus groups and interviews were asked about any challenges they had faced during the intervention in addition to their successes and what had worked well. Three themes were identified in relation to barriers to implementation: poor area reputation and regeneration areas; engaging the local community; and working with LAs. A further three themes were identified in relation to the facilitators for implementation: provision of a coordinator/facilitator; strong local partnerships; and using a range of communication and engagement activities.

#### Barriers

##### Poor area reputation and regeneration areas

The FFW intervention was delivered in deprived areas of England with many having neglected local environments. Participants in focus groups and interviews felt that these areas often had a poor reputation, and this sometimes negatively affected the LAs attitudes towards them:

*“There’s been a lot of change lately, a lot of sort of regeneration in the local area that has involved threats to open space and green space and so on… the council were like, nobody’s interested, they thought they could do what they wanted in the area because it’s generally quite a run down, like a neglected area and they were like, oh well nobody uses the park… and we’re like, no, we do*... *and I think [area] still has that kind of hangover of reputation.” (FG, 2010)*

Some of the communities were located in areas identified for regeneration but participants reported there had been little progress over many years, work had stopped and started, there had been failed promises and much of the community was frustrated and disillusioned. Whilst FFW offered some hope of environmental improvements, it was then difficult to implement these due to uncertainty about existing regeneration plans:

*“While there have been some things that have happened, I mean street signs have gone up and street lights as well, there are a lot of other things that were looked at which really there’s nothing going to happen until the regeneration starts, and we don’t know how long that’s going to be.” (FG, 2011)*

##### Engaging the local community

Engaging local community residents in the intervention activities was considered to be challenging and it was noted that it is often the same, small number of community members who get involved in all local initiatives and activities:

*“The only negativity is getting people, getting the residents to do something, yes some residents do but it tends to be like everything else, it tends to be the same people all the time.” (FG, 2011)*

In contrast, it was thought that individuals in the community would like to get involved but there was a perceived suspicion about, or negative attitudes towards, the project activities proposed. For example, focus group participants indicated that even at the outset some community members expected the environmental improvements (e.g. newly planted flower beds, or maps installed on routes) to be vandalised or destroyed:

*“I think what FFW does is it shows that a lot of people would like to get involved but often it’s something like shyness or suspicion or a feeling of, ‘well whatever we do there’s no point because it will just get destroyed.’” (FG, 2011)*

##### Working with LAs

Working with LAs was reported to take up considerable time and energy pursuing the implementation of environmental improvements. Progress was slow in starting the work required to make the improvements, and there were often delays once the work started, sometimes taking several weeks before work continued and was completed. In some projects, there were issues with obtaining planning permission for the environmental improvements. It was not always clear whether planning permission was needed (e.g. for installing a new notice board) and it took considerable time to find out what was required, and to obtain permission if this was mandatory. This frequently delayed improvements and resulted in very slow progress in some projects. A number of recommendations for improvements identified in street audits were not undertaken by the LA, which community members recognised was due to a lack of funding and budget cuts:

*“It’s having no money that causes the problem and [local authority] are really strapped for cash so they can’t send people out, they don’t have the manpower any more I discovered to come and do any work.” (FG, 2011)*

#### Facilitators

##### Provision of a coordinator/facilitator

All participants in focus groups and interviews highlighted the critical role the coordinators had played in implementing FFW and in facilitating project success. Coordinators were thought to bring leadership and direction to the local project activities: *“It would have been more disjointed one offs I think probably with you know good intention and our efforts, but [coordinator] certainly brought that sense of direction and a strategy really.” (FG, 2011).* They helped groups to link their concerns to other local activities and issues which helped FFW to have a wider impact and become more embedded in the local community. Many focus groups and interview participants indicated that targeting walking had given some of the issues a focus but from a different perspective:

*“We knew what we wanted, but in some aspects we didn’t know how to achieve that so [coordinator] and [local organisation representative] have been an absolute god-send, being able to steer us in the right direction. They’ve actually helped us achieve and now we’ve got a strong committee, we’ve got more members and we’ve achieved such a lot.” (FG, 2011)*

Participants reported that a key role for coordinators was facilitating relationships between communities, and the LA and other local organisations. The coordinators made new contacts, brought people and organisations together and through FFW other local organisations were assisted in achieving their goals. The coordinators also facilitated access to funding, which was provided by the lead organisation or sourced locally. In some communities, the environmental changes which were needed had already been identified but coordinators speeded up progress and provided new ideas for activities, or alternative ways of instigating changes.

Many groups indicated that changes would not have occurred without the coordinator. The coordinators became very involved in communities, regularly attending group meetings and events. They were also important in increasing the involvement of local community members and membership of the registered groups. However, in many projects, groups became reliant on the coordinators such that the groups thought activities would stop if the coordinator withdrew from the project.

Community members identified several personal attributes of the coordinators which they felt aided the success of the relationship and project activities. These included: having knowledge, skills and experience that the community groups did not have, having good communication skills (which helped to keep all stakeholders up to date with progress), being passionate about the local area, being approachable and inspirational, and having expertise in community engagement.

##### Strong local partnerships

Partnerships were thought to play an important role in the success of FFW, particularly when a range of partners were involved:

*“I think where the partnership has worked well is where you’ve had a balance of volunteers, strong partnerships with people with an interest in working in that area, and [coordinator].” (FG, 2010)*

A wide variety of local stakeholders and partners were involved in FFW in addition to the LA. For example, participants mentioned schools, parish councils, local housing associations, police, councillors, local community health partnerships, community payback team, churches, and children’s centres. In addition, other local organisations became involved such as Groundwork, the Bat Conservation Trust and the Wildlife Trust. Developing partnerships was thought to help bring local organisations together for mutual benefit, with FFW supporting other local organisations with similar agendas and in return other organisations supporting FFW. This included the provision of additional funding and resources (e.g. a local landscaping company provided equipment and a team to support environmental improvements). Focus group and interview participants felt their relationship with the LA was enhanced by the FFW project, and links to local stakeholders had been particularly important for extending the reach of the project (e.g. in schools where children had been engaged, this had also filtered home to engage parents in project activities).

##### Using a range of communication and engagement activities

Community engagement and involvement emerged as an important facilitator for the projects. A number of factors were identified which helped to support this. Groups reported using a wide range of methods to communicate information about project activities to local residents including websites, local newsletters, community notice boards, community group contacts, leaflets and posters, and stands at local community events. The street audits also played a role in initial engagement and word of mouth was frequently mentioned as an important method of communication and for engaging new people in activities. Visible action and visible impact were of key importance in engaging community members. Many residents became interested and involved after seeing activities taking place and observing the improvements which had been made:

*“…because they’ve seen the outcome of things, tidier streets and the hanging baskets, they’ve realised well, yeah, it is actually working, we’ll help along, so more people have got involved. At first it was just a very small group of us walking round and like we say we spoke to people on the way round, and of course we all had clipboards and the community police officer was with us and they did want to know what was going on and then more people got involved.” (FG, 2011)*

The importance of communicating with and consulting the local community, and using local knowledge, were highlighted as an important facilitator for successful community engagement and implementation. Participants reported previous experiences of decisions being made by someone who was not based locally and did not understand the local environment:

*“There was a minor issue that somebody’s sat in an office somewhere quite remote from [town], they came up with a plan and said, “this would make a good walk”, and we said, ‘no it won’t, that road’s lethal, nobody’s going to be walking down there’. Just that local knowledge in refining it down to what will work and what is safe and reasonable.” (FG, 2010)*

### Perceptions of intervention impact(s)

Participants were asked about their perceptions of the impact of the project. In most of the communities interviewed, participants reported that FFW had exceeded their expectations. Overall, participants were pleased with improvements made to their local environment, promotional activities, engagement of partners, development of new partnerships and networks, increases in community involvement (particularly intergenerational involvement), reductions in antisocial behaviour and improvements in community cohesion. Only one group indicated they were disappointed with the project. This was due to other local issues related to being in an area identified for regeneration which had impacted on the ability to deliver activities as part of FFW, rather than being due to FFW per se. No other negative effects were reported.

The findings are discussed in relation to the FFW aims: improve the neighbourhood walking environment, encourage communities and local residents to work together to promote walking and increase the number of people walking on specific local routes to key destinations targeted for environmental improvements. An additional theme is discussed which emerged in relation to an unexpected impact: improved social environment.

#### Environmental improvements

There was an overwhelming sense that the environmental changes had improved the local route or area:

*“We’ve cleaned it all up, the bridge has been painted, there’s been new lamp posts put in, and as it’s all near the school it’s just benefited everybody, it’s made such a big difference. I never thought there’d be street lights put up, because it’s always been dark and gloomy. So, they are big benefits, for people’s safety as well as, you know, the general appearance of it, because it was horrible, there was graffiti everywhere and run-down, and it just made kids go under there and drink because it was a mess.’” (FG, 2010)*

Perceived improvements in safety were often highlighted in relation to traffic safety, safety from crime, reduced litter (e.g. used needles, broken glass), removal of street furniture (e.g. railings and bollards) and improved street lighting:

*“A lot of people have commented about it and it’s much safer now because there’s four or five schools all in the vicinity, you know, and people, young people with pushchairs and old people getting off buses from shopping and going to shopping, they don’t have to walk on these busy roads now.” (Interview, 2010)*

*“…because they’ve gone [the planters], you can see now see from one end of street right down, without having to go around things and worrying who’s hiding behind them...” (FG, 2011)*

In addition to the actual environmental changes, participants reported that FFW had impacted on the community and local residents by increasing people’s awareness of the state of the street and walking environment in their own area. Participants reported seeing things they had not taken notice of previously and thought that they were considering things in a different way, from the perspective of walking:

*“[Coordinator] sort of made you aware of things that you’d never thought about before, and it made us start looking at things like lamp posts, rusty lamp posts or a broken window in the street, how it made the area look, start to look run down which actually encouraged vandals and things.” (FG, 2011)*

Participants also mentioned that knowledge and awareness of where to walk in their local areas had increased, and some participants had discovered new areas and features of their local neighbourhoods that they did not know about, despite having lived there for many years:

*“I mean I’ve lived in that area for a long time and I didn’t know that there was like a short cut route on it, and I thought that’s really handy now, I can go to the shop without getting in my car.” (Interview, 2010)*

#### Community working together to promote walking

The project activities were thought to be successful in bringing the community together to undertake intervention activities and promote walking. One of the key impacts mentioned by many groups was the intergenerational and inclusive nature of the activities:

*“It’s been good for families because a lot of it has cascaded down, because originally we did start working with grandparents, and when we did some of the projects, we’d see the grandparents with their children and their children, you know. So, it was good to get the whole family in on something that they could all do together, no matter their ability.” (FG, 2011)*

Most groups indicated that the intervention activities had engaged members of the community and local residents and often those who would not normally engage in community activities got involved, or became a member of the registered community group:

*“I’m quite surprised at the number of people who are more willing to get involved in things now because it was quite closed in the beginning, as you were walking around people would wander away or they’d turn their back, whereas now as you said they’ll come out they’ll ask what you're doing, they’ll want to join in, they’ll want to know about the project.” (FG, 2011)*

#### Increase the number of people walking on local routes

When asked about the impact of the project on walking, few participants mentioned increases in the number of people walking on specific local routes to key destinations targeted for environmental improvements. Instead, many community members discussed participation in the walking activities that had taken place as part of the intervention, such as walking groups or themed walks, or referred to recreational walking and dog walking rather than walking for transport. Some groups noted more people were out on the streets (but did not specifically refer to walking to and from local destinations).

#### Improved social environment

Participants in focus groups and interviews reported substantial changes in the community social environment as a result of FFW. This was an unexpected impact of the intervention. For example, participants indicated that the project had helped to develop community relationships: *“I think a lot of it is from the social point, I think it’s brought a lot of people together from different areas and different backgrounds and different abilities. I think that has helped build up relationships.” (FG, 2010).* These developments were thought to have improved community cohesion and there was a perception that the local areas were much safer as a result: *“The social side, the impact of bringing people together, and bringing agencies together, so it’s promoted partnerships, its promoted community cohesion, inter-generational work. It’s made the places a lot safer, better.” (FG, 2010).* In addition, communities were thought to have become much friendlier places to live in with increased social interactions on the streets: *“We’re getting a lot of strangers... people will be walking up and down the road, they wouldn’t even say good morning before, now we’re getting it, good morning, or they’ll stop and have a chatter.” (FG, 2011).*

Participants in several focus groups mentioned the ownership and sense of pride that had developed for their local areas through the community consultation and following the improvements that had been made. Participants also felt they had started to ‘police’ the local area themselves and take action when needed, rather than wait for someone else to do it:

*“That is really empowering communities when you engage with communities and say, ‘What do you want? Do you want to be involved in that process?’ then people get a sense of ownership and that’s what we’re all about really and if we can extend that out into the wider community.” (FG, 2010)*

*“It’s very much this idea of ownership. If you can identify with an area then you sort of look after it and I think the fact that people are no longer dumping things in the [location] because it looks well cared for, you know it looks as if people are interested whereas in the past I think it became so neglected, whether it’s lack of money or what, but just people thought ‘well you can do it and nobody bothers’.” (FG, 2011)*

It was also thought that, as a result of FFW, more residents were willing to take action rather than disregarding issues, as well as giving the community confidence to question changes that were being made to their local neighbourhood environment without any consultation:

*“We’re actually getting more residents willing to complain about what’s happening in the neighbourhood rather than shut the curtains and shut the door and ignore it.” (FG, 2011)*

*“Now we would question something if something suddenly appeared, you know, or they started digging up a pavement or whatever. It would be questioned now and so that’s given us a kind of confidence to not just to take what’s given to you or what just suddenly appears one day.” (FG, 2010)*

In several projects, FFW activities were thought to have engaged local trouble makers which led to reductions in anti-social behaviour. Participants reported increased use of the streets which also resulted in a perceived reduction in opportunities for anti-social behaviour:

*“If there’s more people on the streets it’s a lot safer, you feel safer than being on your own, so it’s made the area safer as well. If you’re not using your streets, you’re passing them over to the people, the trouble-makers, the drunks, you know, whereas everyone’s now out in them. I think if you don’t use them you lose them.” (FG, 2010)*

Further social environmental benefits which were mentioned included: people coming together to organise and attend community events, tenants in rented properties being more cooperative, and neighbours offering each other mutual help and support.

## Discussion

To date, there has been a paucity of literature examining community experiences of implementation and perspectives of the perceived impact of environmental interventions which aim to promote walking for transport. The aim of this study was therefore to evaluate community perceptions of the barriers and facilitators for intervention implementation and the perceived impact of the FFW intervention. This study adds to evidence regarding the design, development and implementation of interventions which use a community engagement approach to change the physical environment to promote walking for transport and provides insight into the potential impacts of such interventions.

Many of the barriers and facilitators for implementation identified by community members in this study concur with previous research findings reported by intervention developers and deliverers in other walking, physical activity or health interventions. Specifically, in relation to: the challenges of engaging community members [17]; the importance of consulting stakeholders, including meaningful community involvement [28, 36]; the important role of word of mouth in recruiting participants for community-based walking interventions, particularly in ‘hard to reach’ groups [37, 38]; and the need for collaborative partnerships and ongoing support from stakeholders when using community engagement approaches [28, 39]. The critical role of trained facilitators (coordinators) with specific skills and attributes to help deliver the intervention noted in this study has also been highlighted elsewhere [39]. These factors should be taken into consideration when designing the implementation of future interventions.

This study contributes further evidence regarding the challenges of making environmental improvements in areas which may have a poor local reputation or working in areas which are already undergoing regeneration. This may impact on the future selection of target areas for interventions to improve the physical environment with priority given to areas where there is LA support, community involvement and the most substantive and rapid changes can be made. The findings from this study highlight the difficulties for communities of working with LAs, who were sometimes unable to meet the resource and funding demands needed to implement even small-scale environmental changes in a timely manner. This also presents a challenge for researchers, practitioners and policy makers interested in implementing interventions in this field. It has been noted that insufficient resources and a lack of incentives for improving physical environments to promote walking will affect the extent and quality of intervention implementation and evaluation [17]. Therefore, dedicated investment and support from national and local Government is needed to implement this type of intervention. The recent UK Cycling and Walking Investment Strategy outlines a commitment to provide funding for walking (and cycling) infrastructure and to prioritise active modes of transport such as walking in long-term transport planning [40]. However, the extent of implementation and the impact of this strategy is yet to be determined.

**Fig. 1:**
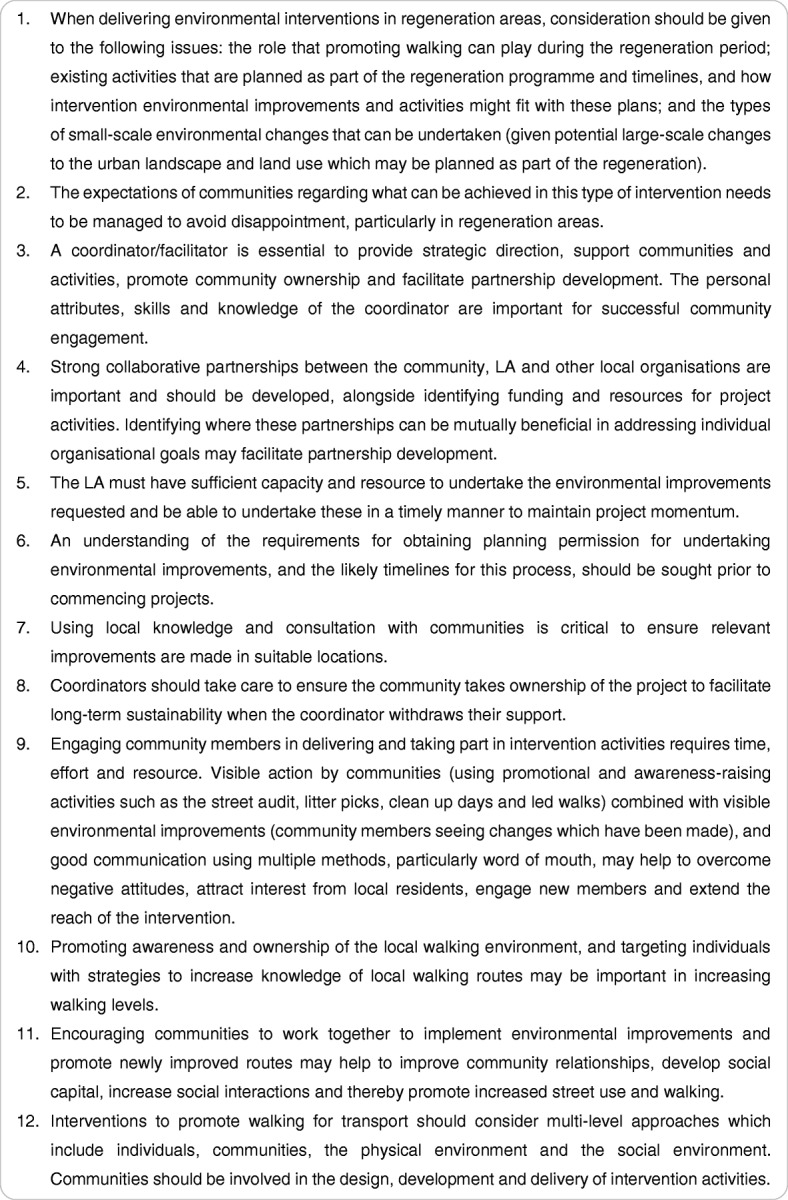
Recommendations for future interventions to promote walking for transport

The aforementioned findings on intervention implementation have important implications for research, practice and policy and in working with communities and local authorities to improve the physical environment to promote walking for transport. Based on the findings from this study, recommendations for the development and delivery of future interventions are provided in Fig. 1. Future research should aim to determine the most effective strategies for utilising community engagement approaches in this type of intervention and should use implementation science methodology [41] to determine the strategies and processes required for optimal intervention delivery. This area of research is particularly important given recent UK guidance for improving the physical environment to promote physical activity, which encourages the use of community engagement approaches and LA action [42].

Participants perceived that the small-scale environmental changes made in the FFW intervention led to improvements in infrastructure and aesthetics, and increased safety from traffic and crime/anti-social behaviour. Changing perceptions of the neighbourhood physical environment may be important for increasing walking for transport as it is known that some environmental attributes are associated with higher levels of transport-related walking [12–16]. The FFW strategy was effective in making some of the small-scale environmental changes which might support walking for transport and thus should be considered as an approach for use in future interventions. Making visible improvements to the environment was thought to be important for providing evidence of successful community action, engaging new community members in intervention activities and sustaining interest. Thus, strategies for increasing visibility and raising awareness of the improvements should also be considered in future, as this has the potential to extend the reach and impact of the intervention. This may be important for influencing walking for transport (and overall physical activity participation) at the community-wide level. In addition to increasing awareness of the environmental changes undertaken, the findings show that promotional and awareness-raising activities (such as led walks) may have an important role to play in changing individual behaviour through increasing knowledge of local routes. The importance and effectiveness of delivering promotional activities, and/or individual behavioural interventions, in addition to making changes to the physical environment, requires further research [42].

Changes in awareness of, and community interest in, the local neighbourhood environment reported as a result of FFW may also be important, as this can encourage and support communities to take action to “influence both political and private sectors by demanding urban planning and design that facilitates walking, cycling and public transport” to help create healthy cities [43]. Thus, the FFW intervention approach may have an important role to play in building community capacity and skills for lobbying LAs to implement improvements to the physical environment in local neighbourhoods, which will help to support increases in walking for transport.

In addition to the physical environment, the social environment is identified in socioecological models as an important influence on physical activity levels [11]. Although changing the social environment was not an aim of FFW, in most communities, participants emphasised social environmental changes resulting from the community working together to implement the intervention (e.g. improved community relationships and cohesion, increased friendliness and increased social interactions on the streets). These types of changes may be significant for promoting walking in local neighbourhoods as associations between positive perceptions of the community (including having a strong sense of belonging, feeling safe and considering the community to be harmonious) and levels of walking have previously been observed [44]. Furthermore, community cohesion has been found to be associated with increased time spent walking for transport [45]. The improvements to the social environment reported by participants suggest the FFW intervention was effective in increasing social capital (including sense of belonging, trust, norms of reciprocity, civic action and social support). Low social capital has been shown to be associated with physical inactivity [46], therefore increasing social capital may be an important target for interventions. Overall, the perceived social environmental changes reported may be an important but unexpected outcome of the intervention strategy used in FFW for influencing walking for transport behaviour.

To date, the relationships between the three constructs of the physical environment, social environment and physical activity levels (including walking for transport) have been relatively under researched. However, highly walkable neighbourhoods are reported to have higher levels of social capital [47–49] and a review of the associations between the built environment and health found that neighbourhoods that are more walkable are associated with increased social capital and increased physical activity [50]. The relationship between the physical and social environments, and the relative importance of changing the social and physical environments for promoting walking for transport, require further examination to inform future intervention design.

In contrast to the other aims of FFW, participants rarely commented on any impact of the intervention on the third aim: increasing the number of people walking on specific intervention routes, although they did mention increased use of the streets, which may be a proxy indicator that more people were walking. The perceived increase in street use may be an important step towards increasing walking for transport in a community by increasing perceptions of safety. This is because increasing the number of people on streets in the neighbourhood can increase natural surveillance through having ‘eyes on the street’ [43] and can make streets appear less threatening [10]. Findings reported previously showed that FFW did result in increases in walking levels/increased use of improved routes [32]. The lack of participants’ discussion regarding walking for transport suggests that this outcome may be of less importance to individuals than improving their local neighbourhood environment or the social aspects of their communities, or that the physical and social environmental aspects had a more noticeable impact on the communities in the short-term. This may have implications for future interventions designed to promote walking for transport. These may need to initially focus communities on improving their local environment and working together on activities and events to promote the improved environment and walking more generally; with increases in walking for transport becoming a longer-term outcome following physical and social environmental changes in the community. Based on the findings in this study, future interventions might consider using a “stealth” approach, whereby the main outcome of interest (in this case walking for transport) is a side effect of the intervention, and the target behaviours for the intervention are related to processes which communities are more motivated by, or place more value on (e.g. improving the local physical and social environment) [51]. This approach warrants further research.

**Fig. 2:**
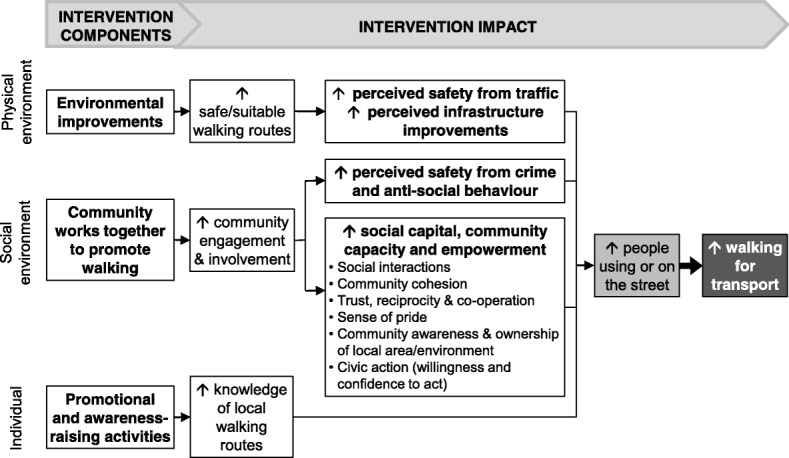
Conceptual framework for a community-based, multi-level intervention strategy to increase walking for transport

The findings from this study suggest that using community engagement approaches, including encouraging communities to work together to develop and deliver intervention activities, can lead to small-scale physical environmental improvements and improvements in the social environment, including social capital. This in turn may result in increased street use and ultimately, increases in walking for transport. Based on these findings, a conceptual framework outlining the approach for a multi-level intervention to increase walking for transport, including targeting individuals, and physical and social environments, is proposed (Fig. 2). Such multi-level interventions based on socio-ecological models require further research to assess their effectiveness on individual behaviour change, and to determine the relative importance of each component (individual, social environment and physical environment), and how the components interact, within different contexts.

### Strengths and limitations

A strength of this study was the number of individuals who took part in the focus groups or interviews which included representatives from the community groups registered to the project and other local residents. None of the community groups or individuals invited refused to participate. The lead researcher (EJA) was independent from the FFW intervention and had no involvement with the community groups prior to or after the focus groups and interviews, minimising bias in the findings. The qualitative approach used in this study enabled a more in-depth investigation of communities’ experiences of implementing the intervention and potential impacts. Such approaches have been highlighted as being important for understanding communities and complex public health problems, in addition to the quantitative approaches traditionally utilised [52]. The findings may be transferable to other areas; however, further research is needed to explore the impact of the FFW approach in different contexts, for example, in areas with differing levels of social capital and walkability. Limitations include that it was only possible to undertake interviews or focus groups in 19 of the 155 FFW communities due to budgetary constraints. The community projects were selected to take part in focus groups and interviews based partly on progress that had been made in projects, therefore they may not have been representative of the other communities involved in FFW. They were however recruited from diverse geographical areas to try and ensure representation from different contexts.

## Conclusion

This study adds important community perspectives of the barriers and facilitators influencing the implementation of an intervention to promote walking for transport. Barriers included: poor area reputation and regeneration areas; engaging the local community; and working with local authorities. Facilitators included: provision of a coordinator/facilitator; strong local partnerships; and using a range of communication and engagement activities. Based on these findings, recommendations for the design and implementation of future interventions are provided and should be considered by researchers, practitioners and policy makers. Using community engagement approaches and encouraging communities to work together can lead to perceived improvements in the physical and social environment resulting in increased street use, which may increase walking for transport. Future research should seek to understand the most effective ways to engage communities in improving physical environments to promote walking for transport, determine optimal implementation strategies, and should investigate the relative importance of improving the physical environment versus the social environment, how improvements to both these environments might interact to maximise intervention impact, and whether individual behaviour change strategies are also required to increase walking for transport.

## Additional files

Additional file 1: Discussion guide for focus groups and interviews. (DOCX 21 kb)

Additional file 2: Summary of community projects who took part in focus groups/interviews. (DOCX 30 kb)

## Abbreviations

FFW: Fitter for Walking
LA: Local authority
