# Supplementary material for: Community perceptions of the implementation and impact of an intervention to improve the neighbourhood physical environment to promote walking for transport: a qualitative study
Source: BMC Public Health. 2018 Jun 8;18:714. doi: 10.1186/s12889-018-5619-y (PMC5994047; doi:10.1186/s12889-018-5619-y)
Supplement: Supplementary file 1 — Discussion guide for focus groups and interviews. (DOCX 21 kb) [file 12889_2018_5619_MOESM1_ESM.docx]

**ADDITIONAL FILE 1**

**Table A1 Discussion guide for focus groups and interviews**

| **Theme** | **Question** |
| --- | --- |
| Introduction and context | - Can you tell me about the [registered group]? - Can you tell me about the local area and walking routes? |
| Project purpose and goals | - How did you find out about/hear about Fitter for Walking? - What do you think the main purpose of the project is? - What were the main reasons you wanted to get involved? - What are the barriers to walking in your local community? - Please can you tell me about the route which was selected for the project in your local area? |
| Roles and responsibilities   - Leadership - Role of the registered group - Role of the community - Role of the project co-ordinator - Role of the local authority - Role of other groups | - Overall, who has led or driven the project activities? - For each stakeholder: - What was [the stakeholder’s] role in the project? - How important was it to have them involved? - What were the challenges in engaging other members of the community? - How might these challenges be overcome? |
| Project implementation (activities) | - How has the project involved members of the registered group/community in planning and implementing programme events and activities? - What activities have taken place, or what changes have been made as a result of the project? - What has been good about the project / improvements to walking routes in your community? - What problems or challenges have there been with the project? - What advice would you give to another group starting this type of project? |
| Project impact | - Overall, has the project met your expectations? - Do you think local residents have walked more along the route to local destinations as a result of the project? - What impact has the project had more generally? - Have there been any negative effects of the project? - Has anything unexpected happened as a result of project activities? |
| Sustainability | - Do you think the changes and improvements which have been made will be maintained in the foreseeable future? - What do you think will be needed to ensure this happens? |
| Additional comments | - Do you have any other comments you would like to add about the project? |
