# Supplementary material for: Community perceptions of the implementation and impact of an intervention to improve the neighbourhood physical environment to promote walking for transport: a qualitative study
Source: BMC Public Health. 2018 Jun 8;18:714. doi: 10.1186/s12889-018-5619-y (PMC5994047; doi:10.1186/s12889-018-5619-y)
Supplement: Supplementary file 2 — Summary of community projects who took part in focus groups/interviews. (DOCX 30 kb) [file 12889_2018_5619_MOESM2_ESM.docx]

**ADDITIONAL FILE 2**

**Table A2 Summary of community projects who took part in focus groups/interviews***

| **Registered group** | **Date of**  **registration/**  **completion** | **Project area/route** | **Date of street audit** | **Key intervention activities** |
| --- | --- | --- | --- | --- |
| **LONDON** | | | | |
| Marks Gate Older People's Network | Start: 01/11/08  End: 20/06/10 | A route through the subway linking to Chadwell Heath High Road, the station and St Chads park. | 12/01/09 | - Displayed map of route to Chadwell Heath - Walking corridor created to Chadwell Heath: resurfacing, dropped kerbs, clutter removal, pedestrian signage - Stalls at the school fete, summer festival and walking event to promote better parking and walking promotion. - Bulb and shrub planting - Gardens walk |
| Seven Kings and Newbury Park Residents Association | Start: 01/03/10  End: 21/05/11 | Aldborough Road which provides links between the High Street and multiple tube stations, William Torbit School, Downshall Primary School, local shops/businesses and park. | 06/03/10 | - Front garden of St Johns church created as community green space - Bench and plant lockers installed - Street clean-up - Removal of waste from people's front gardens - Community events |
| **NORTH EAST** | | | | |
| Gateshead Jewish Nursery | Start: 26/05/09  End: 31/10/11 | Routes to schools/nursery, Matov Children's Centre, Jewish Community Centre (Bewick Centre), Coatsworth Road shopping area and Jewish colleges. | 30/06/09 | - New raised crossing area with traffic calming - Removal of street furniture - Widened pavements - Talks and meetings with parents and pupils to encourage walking to school using “Walk on Wednesday” and FFW pledge cards. - Led walks with fathers and children via the Matov Children Centre. - Engagement/information sharing event at the Jewish Community Centre - Children took part in FFW craft activities, which were road safety and walking themed. |
| Local Felling Residents | Start: 04/09/09  End: 23/07/11 | Route to Metro Station, Community Centre, Sunderland Road shops and open space. | 28/09/09 | - Footpaths resurfaced - Lighting columns replaced in back lane - Guard rail alongside allotments either painted or removed - Vegetation alongside fence cut back and Japanese Knot weed in allotment treated - Allotment sites have been tidied up - On-going enforcement action by the council regarding derelict property on Mulberry Street - Enforcement action against the owners of untidy properties - Led walks - A resident has taken on 'adopt a station' - issues related to anti-social behaviour, litter, graffiti etc. |
| Friends of St Lawrence Park | Start: 10/11/08  End: 23/06/10 | The Byker Link – route to St Lawrence Park, the Quayside, Shields Road and leisure centre. | 16/12/08 | - Large scale community clean-up day - Led walks throughout the year - Friends of St Lawrence Park organised nature walks along the Byker Link |
| Trinity Gosforth | Start: 15/10/09  End: 22/09/11 | Existing Gosforth High Street History Walks; routes to schools; access to the Trinity Centre from surrounding residential areas. | Not done | - An interpretation board of the map installed in the park - Local walks - Engagement with primary schools to coincide with Walk to School Week - Local history walking map has been distributed to local employees, the history group, local schools and residents |
| Plains Farm and Humbledon Residents Association | Start: 12/05/09  End: 18/06/10 | Route from residential area to shops and leisure facilities. | 28/08/09 | - Installation of dropped kerbs - Led walks - Walk to School Week activities with Plains Farm Primary School who have also completed a walking themed poster competition and a week-long family walking challenge |
| **NORTH WEST** | | | | |
| Taylor St | Start: 10/09/08  End: ND | Routes around school/children’s centre, church, shops and alternative routes to town. | 27/10/08 | - New Lighting - Extra bollards to prevent traffic entering bridge illegally - Bridge painted with anti-graffiti paint - Community clean up with residents - School involvement in artwork - Led walks with local children - Markings for pavement games |
| Empire Theatre | Start: 01/09/09  End: 01/08/10 | Civic walk surrounding history of Empire Theatre and its relationship with the neighbourhood | 01/12/09 | - Production of self-led walking maps for schools, visitors and general public - Led walks over centenary celebrations |
| Hallith Wood Urban Care & Neighbourhood Centre | Start: 01/01/09  End: 05/05/09 | Route around housing estate, manor house and woodland.  Links with school and health centre. | Not done | - Led walks - Map making session |
| Our Back Field | Start: 03/12/10  End: ND | Field path way linking up two estates.  Route to town and schools. | 28/01/11 | - Major gateway improvements - School artwork class - Fun day and litter pick-ups |
| **WEST MIDLANDS** | | | | |
| Friends of Thimblemill Brook | Start: 28/10/09  End: 27/05/11 | Path along section two (Norman Road) of the Brook which links to local green space, library, swimming centre, shops and  schools. | 23/01/10 | - New dog waste bins have been installed and the unsafe bin has been relocated - Path/pavement have been repaired - Information panel installed - Dropped kerbs on Bowling Green Drive installed - A new second information panel installed along section 3 of the Brook - Community Litter Pick - Monthly health walks set up with Sandwell Strides - Library Fun Day (promoted the new walk and work of the Friends Of group) - Community bulb planting/pledge cards events (bulbs donated by local business) - Attended Uplands Manor Primary School Summer Fayre, Library Fun Day, Picnic in the Park and Bearwood Primary Summer Fayre to promote walking along the Brook, used walking pledge cards - Four monthly led walks - Bearwood Sure Start group: presentation, bulb planting and pledge cards - Wildlife Spotter Sheet devised for use at the Brook by local schools |
| Lanesfield Tenants & Residents Association | Start: 28/01/2009  End: 01/07/10 | Route to Taylor Road Open Space including Nature Reserve and Hilton Hall Recreation Fields. | 17/09/09 | - Led walks - Community clean-up day |
| Weddell Wynd Residents | Start: 20/02/09  End: 16/05/11 | Weddell Wynd Linear Path which leads to shops, sports academy, metro (tram) station, youth centre, fishing pools, canals and an open green space. | 25/09/09 | - Footway maintenance to linear path - Installation of litter bin - Removal of high dropped curb and replaced with flat tarmac - Installation of two benches - Bulb planting |
| Friends of Hexthorpe Flatts Park group | Start: 03/03/09  End: 01/05/11 | Routes in park with many features and facilities.  Route to local school. | Not done | - Noticeboard installed at the entrance to the park - Planting of spring bulbs has taken place in the park with some of the neighbourhood team and pupils from Hexthorpe Primary School - Led walks around park starting at the local school involving parents and local residents - A family fun day with a stall that had FFW literature and pledge cards - Regular walking group established with 2 newly trained walk leaders |
| Latin Gardens/  Emley Drive Area Tenants & Residents Association | Start: 10/09/08  End: 01/07/10 | Route along the Roman Ridge which leads to a local primary school, some shops and the post office. | 10/09/09 | - Improved access path to Roman Ridge - New street signs have been installed and hedges have been cut down around play area - Installed new junior goal posts - Re-siting of a street sign - Repainting of a street sign - Cutting down of bushes around school entrance and around playing field |
| Friends of Martinwells Lake | Start: 26/09/09  End: 01/07/10 | A lake surrounded by some woodland and grassland bordered by a housing estate. | Not done | - Installation of new benches - Boardwalks repaired and painted - Completion of lake path - New entranceways, gates and signage on site - Monthly community clean-ups - Planting of spring bulbs by primary school - Walks: led walk as part of Walking Festival; Lantern walk in November - Project film |
| Cliff Hills Community Action Group | Start: 01/10/08  End: ND | This is the main route to the local shops and bus stop for many residents. | 19/11/08 | - Installation of one dropped kerb crossing points - Extension of a path around a small green space in front of the shops to save people walking in the road - School assembly at Redwood Primary School to raise awareness of improvements and promote local walking - Spring Bulb planting with local school and day centre |
| Chinatown Tenants & Residents Association | Start: 24/07/09  End: ND | Routes to shops, bus stops and to a green space. | 24/07/09 | - 3 planters removed; replaced with boulders to stop cars short-cutting through - Multiple walking and bulb planting events held - Community clean-up day and street clean ups - Pledge cards presented to residents and families using the nearby Children's Centre |

*Data from implementation logs kept by coordinators during the project.

ND= no data
